# Sex differences in stretch-induced hypertrophy, maximal strength and flexibility gains

**DOI:** 10.3389/fphys.2022.1078301

**Published:** 2023-01-04

**Authors:** Konstantin Warneke, Astrid Zech, Carl-Maximilian Wagner, Andreas Konrad, Masatoshi Nakamura, Michael Keiner, Brad J. Schoenfeld, David George Behm

**Affiliations:** ^1^ Department for Exercise, Sport and Health, Leuphana University, Lüneburg, Germany; ^2^ School of Human Kinetics and Recreation, Memorial University of Newfoundland, St. Johns, NL, Canada; ^3^ Department of Human Motion Science and Exercise Physiology, Friedrich Schuller University, Jena, Germany; ^4^ Department of Training Science, German University of Health & Sport, Ismaning, Germany; ^5^ Institute of Human Movement Science, Sport and Health, University of Graz, Graz, Austria; ^6^ Faculty of Rehabilitation Sciences, Nishi Kyushu University, Kanzaki, Saga, Japan; ^7^ Department of Exercise Science and Recreation, Lehman College, Bronx, NY, United States

**Keywords:** female, male, range of motion, muscle thickness, strength capactiy, long-duration stretching

## Abstract

**Introduction:** If the aim is to increase maximal strength (MSt) and muscle mass, resistance training (RT) is primarily used to achieve these outcomes. However, research indicates that long-duration stretching sessions of up to 2 h per day can also provide sufficient stimuli to induce muscle growth. In RT literature, sex-related differences in adaptations are widely discussed, however, there is a lack of evidence addressing the sex-related effects on MSt and muscle thickness (MTh) of longer duration stretch training. Therefore, this study aimed to investigate the effects of 6 weeks of daily (1 h) unilateral static stretch training of the plantar flexors using a calf-muscle stretching device.

**Methods:** Fifty-five healthy (m = 28, f = 27), active participants joined the study. MSt and range of motion (ROM) were measured with extended and flexed knee joint, and MTh was investigated in the medial and lateral heads of the gastrocnemius.

**Results:** Statistically significant increases in MSt of 6%–15% (*p* < .001–.049, d = 0.45–1.09), ROM of 6%–21% (*p* < .001–.037, d = 0.47–1.38) and MTh of 4%–14% (*p* < .001–.005, d = 0.46–0.72) from pre-to post-test were observed, considering both sexes and both legs. Furthermore, there was a significant higher increase in MSt, MTh and ROM in male participants. In both groups, participants showed more pronounced adaptations in MSt and ROM with an extended knee joint as well as MTh in the medial head of the gastrocnemius (*p* < .001–.047). Results for relative MSt increases showed a similar result (*p* < .001–.036, d = 0.48–1.03).

**Discussion:** Results are in accordance with previous studies pointing out significant increases of MSt, MTh and ROM due to long duration static stretch training. Both sexes showed significant increases in listed parameters however, male participants showed superior increases.

## 1 Introduction

Strength capacity is of paramount importance in various health- and performance-related settings, with benefits for daily life, good health, and longevity (Cooper et al., 2010; Westcott, 2012; Williams et al., 2017), rehabilitation (Stevens et al., 2004; Maestroni et al., 2020), and performance level in recreational- and elite sports (Styles et al., 2016; Suchomel et al., 2016). Traditionally, resistance training (RT) is the most commonly employed intervention to enhance maximal muscle strength (MSt) (Kraemer et al., 2002) and induce muscle hypertrophy (Schoenfeld et al., 2019; Schoenfeld et al., 2017).

Alternatively, animal research using long-durations of static stretching have demonstrated significant increases in MSt, thickness (MTh) and muscle length (Warneke et al., 2022a). Research using stretching durations from 30 min to 24 h per day, 7 days per week applied to the wings of chickens and quails showed a dose-response relationship for morphological adaptations (Frankeny et al., 1983; Bates, 1993). However, using quails, Bates (Bates, 1993) showed that using 1 hour of stretch led to an increase of 59% in muscle mass (MM), while doubling the stretching duration led to an enhancement of 67%, pointing out a most economical stretching duration of 30 min to 1 h. In contrast, Nunes et al. (Nunes et al., 2020) reviewed the current human literature, showing no significant hypertrophy effects in humans with stretch training. The eligible studies in this review investigated the effects of approximately 2 minutes of stretching per session, which cannot be compared with the much greater stretch durations from animal studies.

However, longer durations of individual static stretching interventions (sessions) with humans of up to 2 hours per day showed significant increases in MSt, which were accompanied by significant hypertrophy (Warneke et al., 2022b). Hence, it is shown if stretching is performed with sufficient session duration, intensity, and weekly frequency flexibility adaptations can be accompanied by increases in muscle hypertrophy and MSt (Warneke et al., 2022b; Warneke et al., 2022c). These studies showed that long duration static stretching sessions implemented for 6 weeks resulted in moderate to large magnitude MSt increases of 14.2%–22.3% (d = 0.51–0.91), increases in MTh and ROM of 15.3% (d = 0.84) and 13.2%–27.3% (d = 0.47–0.87) dependent on used knee joint angle, respectively (Warneke et al., 2022b; Warneke et al., 2022c). Interestingly, previous studies also showed significant cross-education effects in response to stretching interventions of up to 12 weeks regarding MSt (Nelson et al., 2012; Warneke et al., 2022b; Warneke et al., 2022c), which might be attributed to neuronal adaptations induced by increased activity of stretch reflex afferents (Zhou, 2000; Zhou et al., 2022).

A research question, which was not considered in previous research was the sex-related influence on stretch-induced adaptations in MSt, MTh and ROM. A limitation of the aforementioned studies is the lack of calculation of sex differences between groups (Lacio et al., 2021). Even if scientific research showing similar responses in hypertrophy and MSt increases between male and female participants (Roberts et al., 2020) it is a popularly held belief that females show lower adaptations to RT stimuli than males (Lewis et al., 1986). In terms of flexibility, there might be evidence for significantly better baseline ROM values in females compared to males (Cipriani et al., 2012; Yu et al., 2022). However, both, Cipriani et al. (Cipriani et al., 2012) and Yu et al. (Yu et al., 2022), failed to show significant differences between sexes in changes of flexibility due to stretch training (4 weeks, three to six times per week). In this regard, Yu et al. (Yu et al., 2022) countered that a significantly higher passive muscle stiffness was accompanied by significantly lower flexibility in male participants compared to their female counterparts (in pre-test values). Since Morrison et al. (Morrison et al., 2015) showed that Achilles tendon stiffness seems to be influenced by MSt instead of sex, one could question the magnitude of the effects of sex on differences in flexibility. While it is still assumed that male participants show higher absolute MSt and greater muscle cross-sectional area (Nonaka et al., 2006; Nagai et al., 2020), which is often attributed to the difference between sexes in testosterone (Handelsman et al., 2018), the differences in strength capacity seem to disappear when normalized for fat-free body mass (Freilich et al., 1995; Nonaka et al., 2006; Sandbakk et al., 2018). Moreover, a meta-analysis performed by Roberts et al. (Roberts et al., 2020) showed no significant differences in hypertrophic response to RT in the lower extremity between sexes, but even higher effects in female participants in the upper body, which was attributed to lower pre-test training status of the female participants. Accordingly, Bishop et al. (Bishop et al., 1989) stated that there was no difference between trained male and female swimmers regarding their fat-free cross-sectional area, using fat free mass as a covariate.

Based on this premise, the purpose of this study was to investigate the effects of long-duration unilateral and daily static stretching for 1 hour a day over 6 weeks on MSt, MTh and ROM by considering potential sex differences, using a calf muscle stretching orthosis. Furthermore, significant higher increases in MSt, MTh and ROM training adaptations are hypothesized in males compared with females.

## 2 Methods

### 2.1 Study design

Existing data sets (Warneke et al., 2022b; Warneke et al., 2022c) were used to investigated sex-related responses of the plantar flexors to 6-week daily stretching training. Participants were divided into male and female groups. All participants performed 1 hour of unilateral daily stretching with their dominant leg for 6 weeks. Maximum isometric strength, and ankle dorsiflexion flexibility with extended and flexed knee joint as well as MTh were examined in pre- and post-tests. Prior to testing, participants performed a five-minute warm-up consisting of ergometer cycling with 1 W/kg bodyweight.

### 2.2 Participants

A priori calculation of sample size using G-Power revealed a required total sample size of at least 40 participants assuming an effect size of 0.7 based on a previous study in the topic (Warneke et al., 2022), including four groups (intervened leg and contralateral leg from male and female participants) and two measurements. To offset possible dropouts, 55 participants (male: n = 28, age: 27.3 ± 4.1 years, height: 178.5 ± 4.4 cm, weight: 82.5 ± 4.1 kg; female: n = 27, age: 26.9 ± 2.1 years, height: 167.3 ± 3.9 cm, body mass: 65.3 ± 3.3 kg) were recruited in the northern area of Germany for this study. Participants were categorized as athletically active, having performed strength training for at least 1 year in local sports clubs for a minimum of twice a week. Moreover, they had to be free of injury for the last 6 months. All participants were informed about the experimental risks and provided written informed consent to participate in the present study. Approval for this study was obtained from the institutional review board (Carl von Ossietzky University Oldenburg, No. 121–2021). The study was conducted in accordance with the Helsinki Declaration. All participants finished the studies without missing more than two stretching sessions.

### 2.3 Testing procedure

**FIGURE 1:**
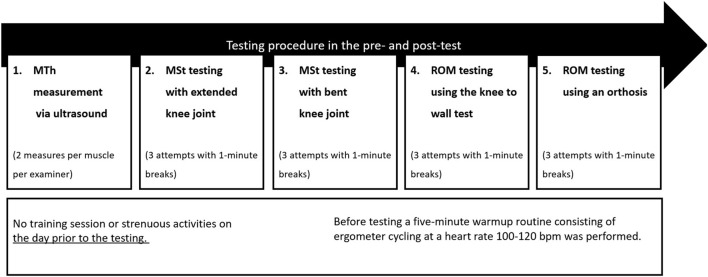
Graphical illustration of the testing procedure.

Warm-up was included before starting the strength testing procedure performing ergometer cycling for 5 min using a heart rate of 100–120 bpm. Testing procedure is illustrated in Figure 1.

MThL = Measurement of muscle thickness in the lateral head of the gastrocnemius, MThM = Measurement of muscle thickness in the medial head of the gastrocnemius, MVC180 = Maximal voluntary contraction testing with extended knee joint, MVC90 = Maximal voluntary contraction testing with bent knee joint, KtW = dorsiflexion range of motion testing using the knee to wall test, ORTH = Dorsiflexion range of motion testing using the goniometer of the orthosis.

#### 2.3.1 Maximal strength testing

**FIGURE 2:**
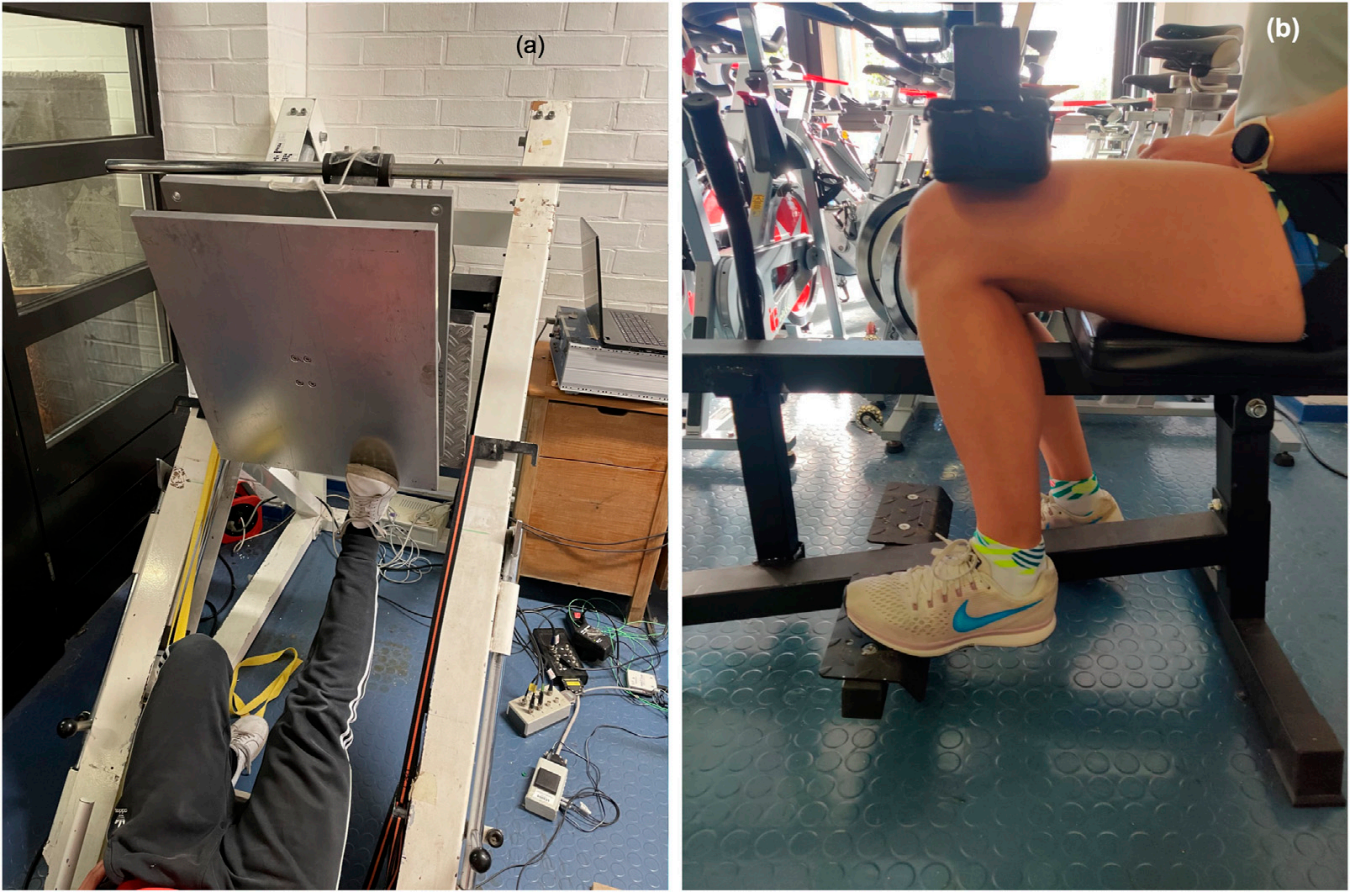
Measurement of MSt in the extended knee joint **(A)** and the flexed knee joint **(B)**.

The testing procedure as well as the devices were used as previously described (Warneke et al., 2022b; Warneke et al., 2022c). Plantar flexors MSt was determined *via* an isometric maximal voluntary contraction (MVC) at a 180° (MVC180) and 90° knee angle (MVC90) under unilateral testing conditions in both the intervened leg (IL) and the control leg (CL) in all participants. A 50 × 60 cm Kistler force plate with a force transducer (company AST, Leipzig, model KAC) with a measurement range of ±5000 N as well as a 13-bit analog-to-digital converter attached to the sled of a 45° leg press was used for examining MVC180. For the MVC180, industrial grade tensioning straps fixed the position so that the starting position was set to an ankle joint angle of 90° with the metatarsophalangeal joint of the foot placed flush to the edge (see Figure 2A). MVC90 was tested using a calf muscle testing device (CMD) equipped with 10 × 10 cm force plates attached to the footrests and force sensors “Kistler Element 9251” with a resolution of 1.25 N, a pull-in frequency of 1,000 Hz, and a measurement range of ±5000 N. A charge amplifier Type5009 and a 13- bit analog-to-digital converter NI6009 were used to record the vertical forces (Fz). Similarly, for MVC90 the straps fixed the thigh pad in a position with the starting position that enabled an ankle joint angle of 90° with the metatarsophalangeal joint of the foot placed flush to the edge (see Figure 2B). Participants performed an isometric MVC against the force plate in response to an acoustic signal and held the contraction for 3 seconds. Participants rested for 1 minute between repetitions to avoid fatigue. Measurements were conducted until no improvement in MSt was recorded, but for a minimum of three trials. The intraclass correlation (ICC) for isometric strength measurements was previously stated with ICC = 0.95 (Warneke et al., 2022c). Reliability of the MSt measurements is provided in Table 1.

**TABLE 1:** Intraclass correlations coefficients (ICC) and the coefficient of variability (CV) are stated to determine the reliability of included tests.

Parameter	ICC (95% CI)	CV (95% CI)
MVC180	0.997 (0.994–0.999)	0.98% (0.84–1.11)
MVC90	0.987 (0.950–0.993)	2.31% (1.92–2.94)
KtW	0.944 (0.925–0.967)	2.97% (2.24–3.29)
ORTH	0.991 (0.980–0.994)	1.11% (1.00–1.64)
MThL	0.913 (0.892–0.929)	3.31% (2.81–4.02)
MThM	0.947 (0.903–0.968)	2.90 (2.22–3.51)

MVC180 = maximal voluntary contraction in the plantar flexors with extended knee joint, MVC90 = maximal voluntary contraction in the plantar flexors with bent knee joint, KtW = ROM, of the ankle joint using the knee to wall test, ORTH = ROM, of the ankle joint using the goniometer of the orthosis, MThL = muscle thickness of the lateral head of the gastrocnemius, MThM = muscle thickness of the medial head of the gastrocnemius.

#### 2.3.2 ROM measurement

**FIGURE 3:**
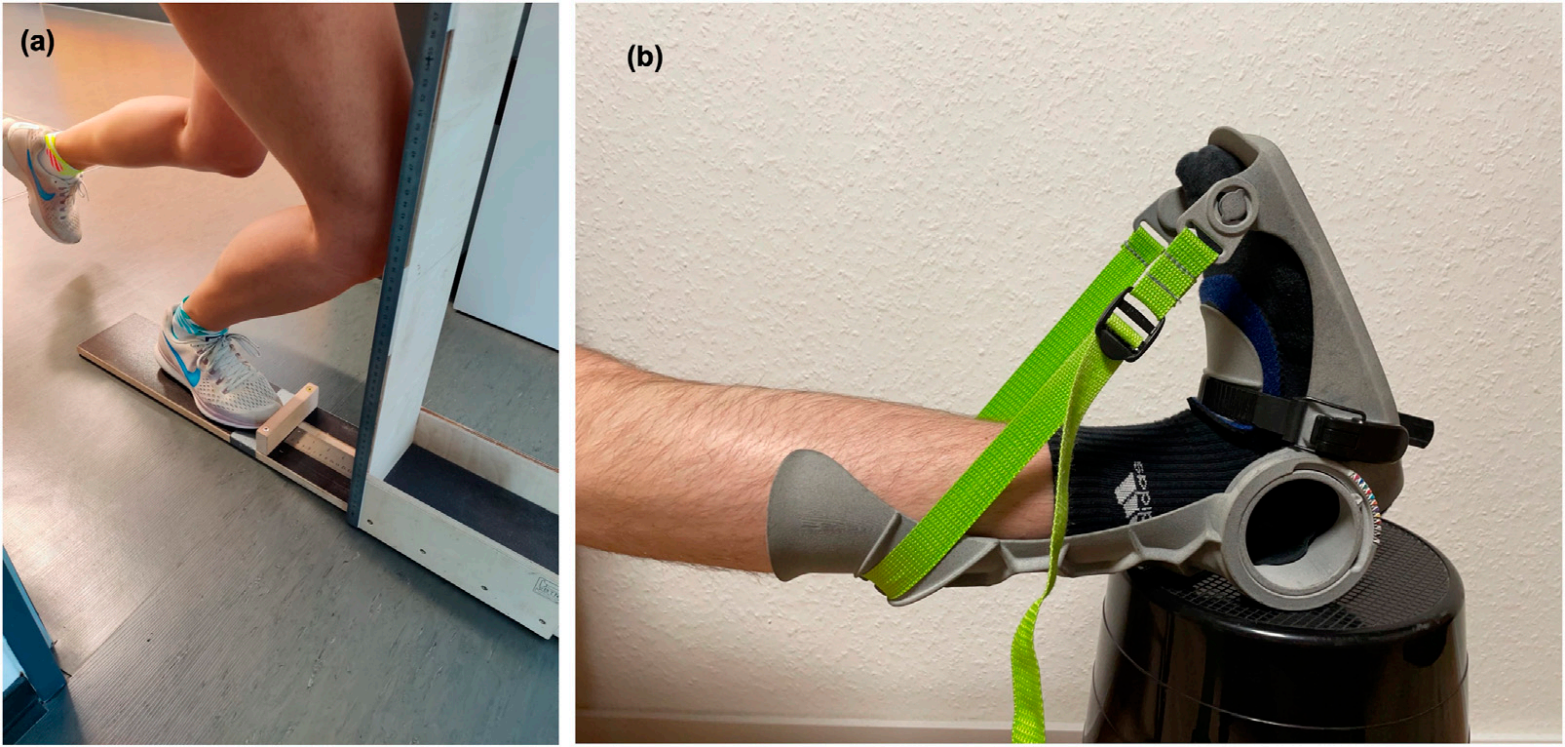
Measurement of flexibility *via* KtW **(A)** and ORTH **(B)**.

ROM in the upper ankle joint was assessed using the “knee-to-wall stretch” test (KtW) and the angle measurement device on the orthosis (ORTH). To use the KtW, a sliding device was used as previously described (Warneke et al., 2022c) to measure ROM in the upper ankle with a bent knee joint. Participants were instructed to place the foot on the attached marker of the device and to push the board of the sliding device forward with their knee until the heel of the standing leg started to lift off (see Figure 3A). During this procedure, one investigator constantly pulled on a sheet of paper. The measurement was stopped when the paper could be pulled from underneath the heel. The value for ROM was obtained from the measuring device in cm. Participants had to perform three valid trials per leg. The best (maximal) value was used for the statistical analyses.

Furthermore, ROM with an extended knee joint was assessed using ORTH. For this purpose, the foot of the participant was placed on an object with the same height as the chair to improve stretching of the plantar flexors and dorsal muscle chain (see Figure 3B). The orthosis was used to reach maximal dorsiflexed position in the ankle joint while staying in an extended knee joint position. Testing started from a neutral 0° position in the ankle and was performed three times. The best trial was used for statistical analysis. Each major indentation of the goniometer corresponds to an increase of 5° and each minor indentation to an increase of 2.5°. With ICC = .99 ROM assessments in the ankle joint using these devices can be classified as high (Warneke et al., 2022c). Reliability of the ROM measurements are stated in Table 1.

#### 2.3.3 Ultrasonography for assessing muscle thickness

**FIGURE 4:**
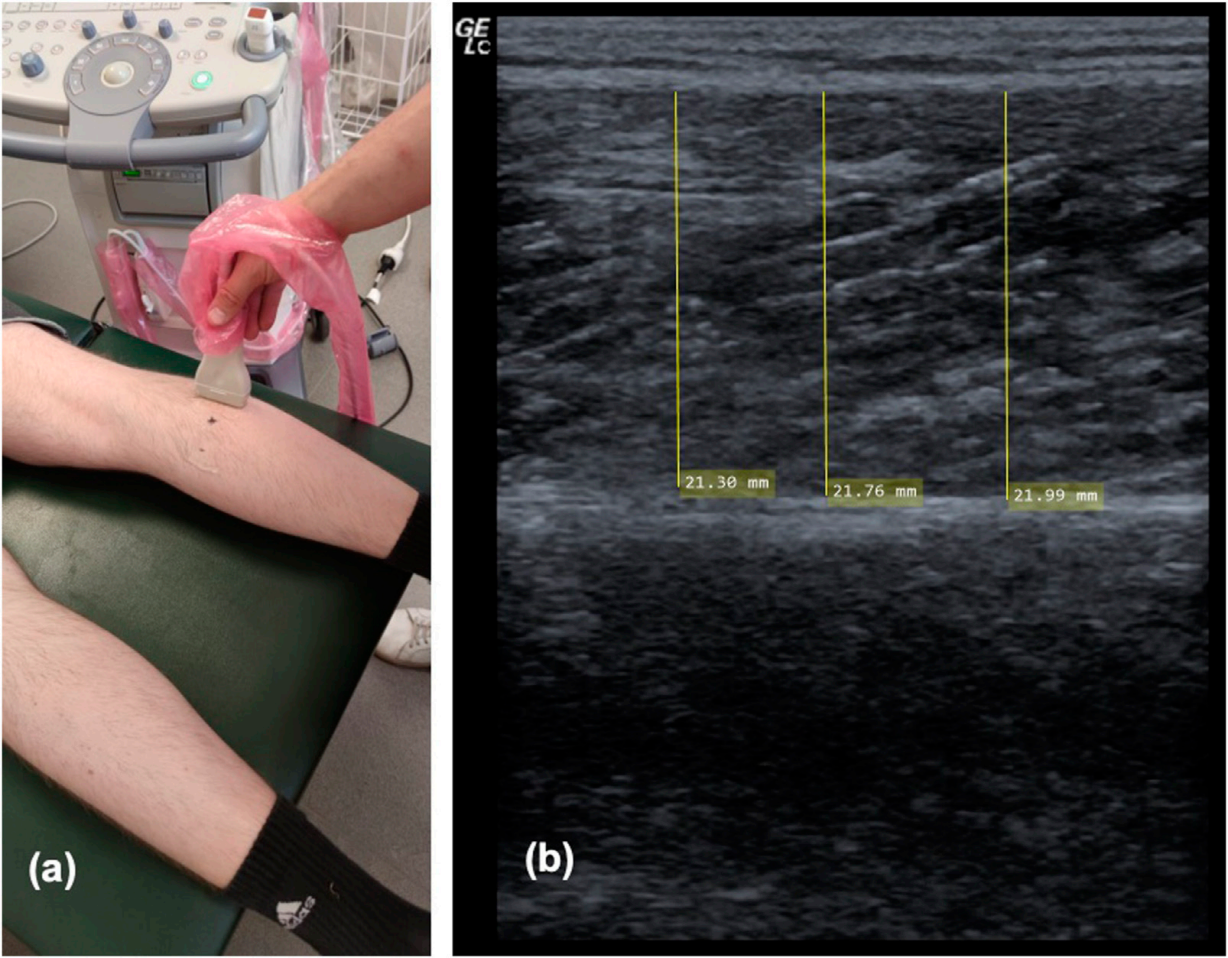
Showing ultrasound procedure on the lateral head of the gastrocnemius **(A)** and one example of sonography measurement of the medial head of the gastrocnemius **(B)**.

As previously described (Warneke et al., 2022c), ultrasonography was used to assess MTh, herein defined as the distance between upper and the deep fascia. Measures were obtained at pre- and post-study in both legs separately, using a two-dimensional B-mode ultrasound with a linear transducer (12, 13 MHz, Mindray Diagnostic Ultrasound System of 5 cm probe length). MTh was determined in the lateral (MThL) and medial (MThM) heads of the gastrocnemius. For this assessment, each participant laid in a prone position with their legs completely extended and feet hanging off the end of a table; they were instructed to stay completely relaxed during imaging. Measurements were obtained at 25 percent of the distance between the most lateral point of the joint space of the knee and the most lateral tip of the lateral malleolus. The superficial and deep aponeuroses were as parallel as possible to optimize visibility of the fascicles as continuous striations from one aponeurosis to the other. For measurement of MTh, the transducer was positioned at the midpoint of each muscle belly perpendicular to the long axis of the participant’s leg. The data for each measurement resulted from averaging three measurements across the proximal, central and sital portion of the produced image (Sarto et al., 2021) (see Figure 4). Two examiners performed two measurements per muscle independently from each other, blinded for the group. Thus, muscle thickness was evaluated by using MicroDicom (Sofia, Bulgaria). Reliability of sonography was determined previously with ICC = 0.95–0.97 (Warneke et al., 2022c).

### 2.4 Intervention

Stretching was performed as previously described (Warneke et al., 2022c). Participants stretched the plantar flexors for 1 hour per day for 6 weeks using a calf muscle stretching orthosis (see Figure 3B). The participants sat with their backs as straight as possible against the backrest of a chair with their foot in the orthosis on a support object of the same height to ensure extension of the knee and optimize stretch of the plantar flexors. The set angle of the orthosis as well as the time of daily stretch was documented by the participants in a stretching log. They were instructed to reach an individual stretching pain of 7-8 on a visual analogue scale of 1–10 (with 0 = no pain; 10 = maximum point of discomfort) at the start of the stretching, the maximum angle could be read from the angle measuring device of the orthosis. Without re-adjusting the orthosis, stretch pain decreased within seconds to few minutes, probably because of assumed relaxation effects. Therefore, the stretching pain was only very high at beginning of the investigation. The participants should perform 1 hour of stretching without any breaks. The stretch stimulus was not to be adjusted, even if stretch pain decreased after a couple of minutes.

### 2.5 Data analysis

The data analysis was performed with SPSS 28. Data are presented using mean (M) ± standard deviation (SD). Normal distribution was confirmed for the used data *via* the Kolmogorov Smirnov test. The best performance in each test were used for the statistical analysis. Reliability was determined using the ICC, 95% confidence interval (95% CI) and coefficient of variability (CV) for listed monitoring assessments (Table 1). Moreover, the Levene-test for homogeneity in variance was performed. The IL and CL of all participants were included for further calculation. One-way ANOVA was used to evaluate significant baseline differences between the groups. Since the one-way ANOVA revealed significant pre-test differences between male and female participants, pre-test values were set to 100% to compare the increases of the different groups post-exercise values were normalized to pre-test values. A mixed model two-way ANOVA with repeated measurements was used for transformed values and Time effects as well as Group*Time interactions. To investigate if there was a significant percentage increase from pre-to post-test, paired t-tests were used and corrected for α-error using the false discovery rate by Benjamini—Hochberg (Ferreira and Zwinderman, 2006) as *post hoc* analysis for time effects. The Scheffé-test was used as a *post hoc* test for mean differences of increases between groups to calculate significant differences in increases between sex and legs. Effect sizes are presented as Eta squares (ƞ^2^) and categorized as: small ƞ^2^ < 0.06, medium ƞ^2^ = 0.06–0.14, large ƞ^2^ > 0.14 (Cohen, 1988). Additionally, effect sizes are reported with Cohen’s d (Cohen, 1988) and categorized as: trivial <0.2, small d = 0.2−<0.5, medium d = 0.5–0.8, large d > 0.8. Since literature indicates that there might be differences in results using relative strength values compared to absolute strength values, relative strength values (absolute value/body mass) were also used for calculation. Post-hoc power (1-β) was calculated *via* G-Power (Version 3.1, Düsseldorf, Germany). The level of significance was *p* < .05.

## 3 Results

### 3.1 Assessment of sex-related baseline differences

There were significant differences between males and females in pre-test values for MVC180 (*p* < .001), MVC90 (*p* < .001) and ORTH (*p* = .011), showing higher values in male participants in strength-related parameters, while females showed higher flexibility. No sex-related differences were observed for KtW (*p* = .552), MThL (*p* = .297) and MThM (*p* = .239) when considering absolute values. With relative values, there was no difference in pre-test values for MVC180 (*p* = .419). One-way ANOVAs determined significantly higher values (*p* < .001) for strength MVC90, MThL and MThM in male participants. However, females had significantly higher flexibility values.

### 3.2 Assessment of sex-related changes from pre-to post-test

**FIGURE 5:**
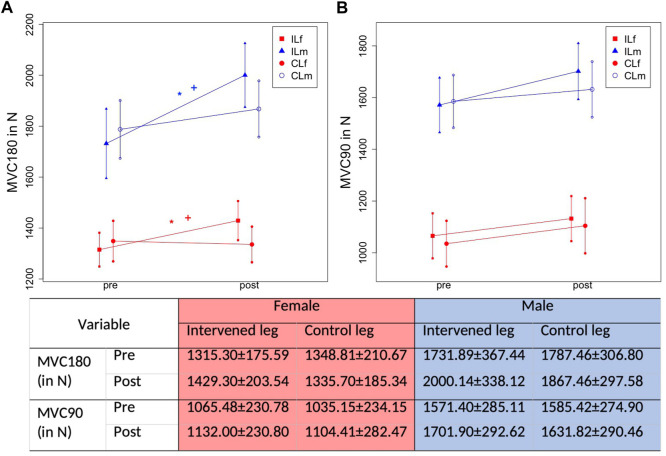
Graphical illustration of progression in ROM in IL and CL due to 1-h daily stretching training in plantar flexor in MVC180 (Maximal strength testing with extended knee joint) **(A)** and MVC90 (Maximal strength testing with bent knee joint) **(B)** considering sex. The * illustrates a significant difference to the control leg, the + illustrates a significant difference to the other sex.

**FIGURE 6:**
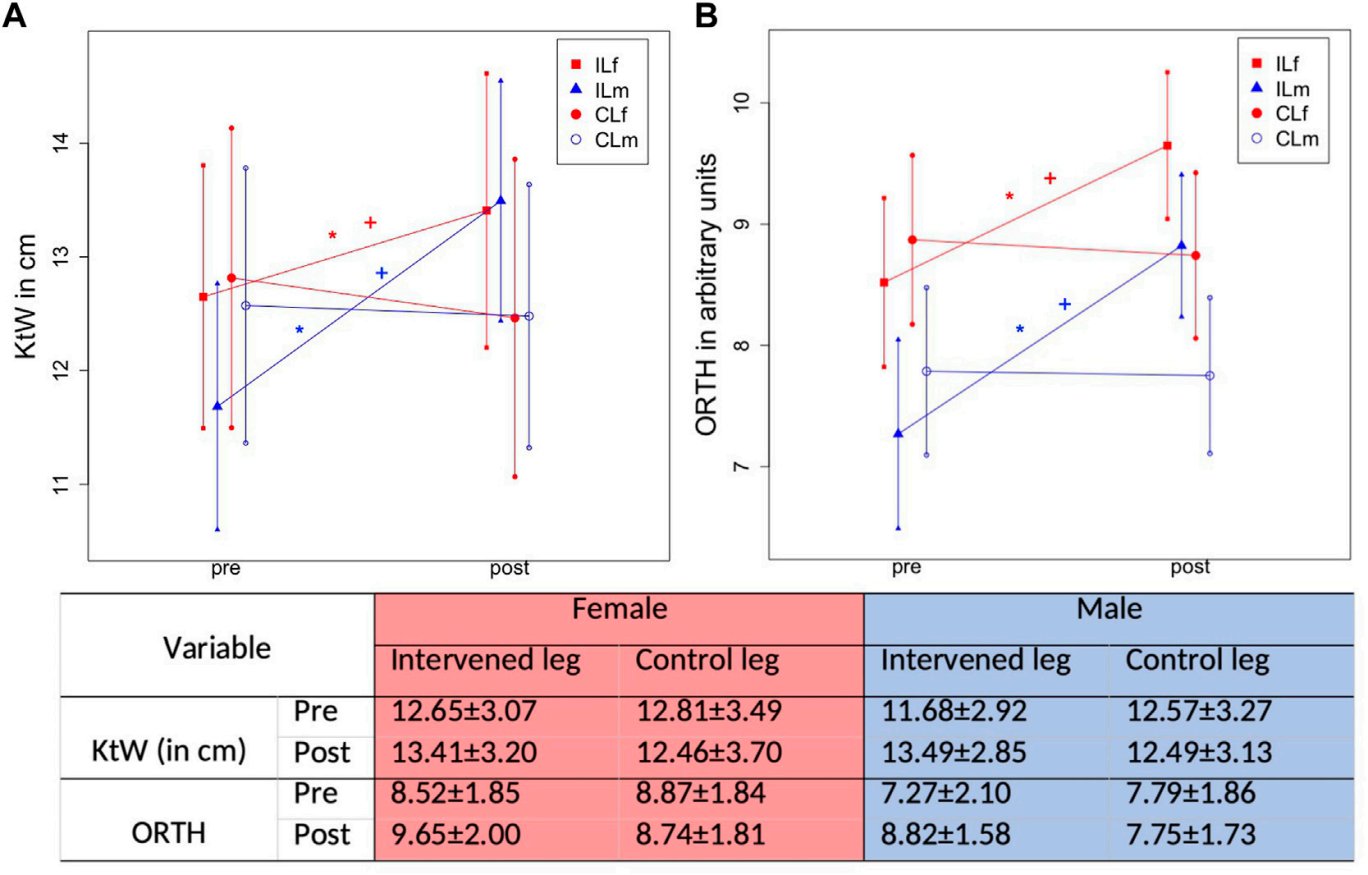
Graphical illustration of progression in ROM in IL and CL due to 1-h daily stretching training in plantar flexor in KtW (ROM testing using the knee to wall test) **(A)** and ORTH (ROM testing using the goniometer of the orthosis) **(B)** considering sex. The * illustrates a significant difference to the control leg, the + illustrates a significant difference to the other sex.

**FIGURE 7:**
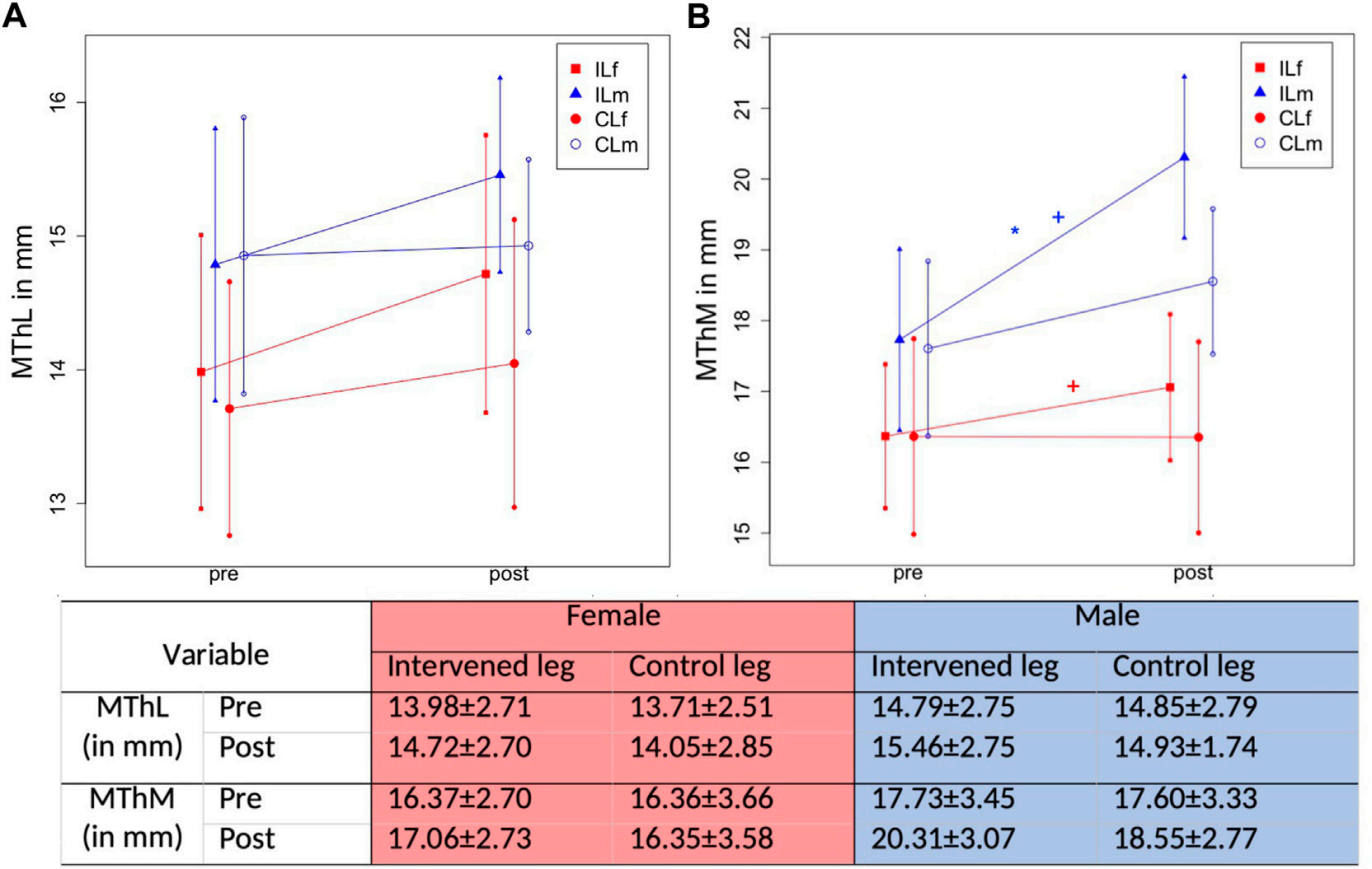
Graphical illustration of progression in MTh in IL and CL due to 1-h daily stretching training in plantar flexor in MThL (muscle thickness in the lateral head of the gastrocnemius) **(A)** and MThM (muscle thickness in the medial head of the gastrocnemius) **(B)** considering sex. The * illustrates a significant difference to the control leg, the + illustrates a significant difference to the other sex.

Mean stretching time per week was 6.7 ± 0.8 h. Progression of absolute values for MVC180 and MVC90 are displayed in Figure 5, progression of KtW and ORTH are displayed in Figure 6 and progression of MThL and MThM are illustrated in Figure 7. Table 2 provides the percentage values in the post-test when compared to the pre-test, as those were set to 100%.

**TABLE 2:** Post-test percentage values of included parameters in relationship to the pre-test 100%.

Parameter	Intervened leg		Control leg	
	Female	Male	Female	Male
MVC180 (in %)	8.7 ± 8.76^a^ ^,^ ^b^ *p* < .001^c^	15.5 ± 10.53^a^ ^,^ ^b^ *p* < .001^c^	−1.0 ± 8.86 *p* = .574	4.5 ± 7.01 *p* = .002^c^
MVC90 (in %)	6.2 ± 10.76 *p* = .006^c^	8.3 ± 10.6 *p* < .001^c^	6.7 ± 20.29 *p* = .098	2.9 ± 7.95 *p* = .062
KtW (in %)	6.0 ± 9.20^a^ ^,^ ^b^ *p* = .002^c^	15.5 ± 6.33^a^ ^,^ ^b^ *p* < .001^c^	−2.75 ± 8.30 *p* = .097	−0.73 ± 7.10 *p* = .587
ORTH (in %)	13.3 ± 11.00^a^ ^,^ ^b^ *p* < .001^c^	21.4 ± 13.02^a^ ^,^ ^b^ *p* < .001^c^	−1.5 ± 8.30 *p* = .355	−0.46 ± 7.40 *p* = .745
MThL (in %)	5.2 ± 8.87 *p* = .005^c^	4.5 ± 11.38 *p* = .044	2.5 ± 12.35 *p* = .309	0.5 ± 13.11 *p* = .841
MThM (in%)	4.2 ± 9.10^b^ *p* = .023^c^	14.5 ± 11.76^a^ ^,^ ^b^ *p* < .001^c^	−0.01 ± 11.61 *p* = .976	5.3 ± 14.55 *p* = .06

MVC180 = maximal voluntary contraction in the plantar flexors with extended knee joint, MVC90 = maximal voluntary contraction in the plantar flexors with bent knee joint, KtW = ROM, in the dorsiflexion using the knee to wall stretch, ORTH = ROM, in the dorsiflexion using the goniometer of the orthosis, MThL = muscle thickness in the lateral head of the gastrocnemius, MThM = muscle thickness in the medial head of the gastrocnemius.

^a^ significant difference compared to the control leg

^b^ significant difference compared to the other sex

^c^ significant percentage increase from pre-to post test.

### 3.3 Measurement of maximal strength

For MVC180 absolute strength values, there was a time effect (F1,106 = 66.64, *p* < .001, ƞ^2^ = 0.39) and a group*time interaction (F3,106 = 16.84, *p* < .001, ƞ^2^ = 0.32). The Scheffé test revealed higher increases in the male IL compared with female IL (*p* = .049, d = 0.45) as well as higher improvements in the female IL compared with female CL (*p* = .002, d = 0.63). Furthermore, there was a significantly higher enhancement in MVC180 for the male IL compared with all other groups (*p* < .001–.049, d = 0.74–1.09). No differences were found for the progressions between the female IL and male CL (*p* = .387) and the female CL and male CL (*p* = .167).

For MVC90 absolute values there was a time effect showing MVC increases (F1,106 = 23.06, *p* < .001, ƞ^2^ = 0.18) but no significant group*time interaction (F3,106 = 0.82, *p* = .486, ƞ^2^ = 0.02).

There was a time effect (F1,106 = 67.69, *p* < .001, ƞ^2^ = 0.39) and a Group*Time interaction (F3,106 = 16.67, *p* < .001, ƞ^2^ = 0.32). The Scheffé test showed a higher increase in the male IL compared with female IL (*p* = .036, d = 0.48) and higher increases in the female IL and CL (*p* = .003, d = 0.54). Furthermore, there were higher increases in the intervened leg of the male participants compared with the CL of male and females (*p* < .001, d = 0.72–1.03).

With MVC90, there was a significant increase associated with a time effect (F1,106 = 25.83, *p* < .001, ƞ^2^ = .196), but no significant Group*Time interaction (F3,106 = 1.026, *p* = .384, ƞ^2^ = .028).

### 3.4 Measurement of flexibility

A Time effect (F1,106 = 36.75, *p* < .001, ƞ^2^ = 0.26) and Group*Time interaction (F3,106 = 30.94, *p* < .001, ƞ^2^ = 0.47) was observed for ROM *via* the KtW. The Scheffé test revealed significantly greater increases in the male IL compared with female IL (*p* < .001, d = 0.717) and also higher increases for the females IL versus CL (*p* = .001, d = 0.65). There was a higher increase in the male IL compared with all other groups (*p* < .001, d = 1.24–1.38). No difference was shown in the change of both CLs between male and female participants (*p* = 0.822).

For ORTH there was also a significant increase with a time effect (F1,106 = 71.63, *p* < .001, ƞ^2^ = 0.40) and Group*Time interaction (F3,106 = 33.02, *p* < .001, ƞ^2^ = 0.48). The Scheffé test revealed higher increases in the male IL compared with the female participants (*p* = .037, d = 0.47) and for the IL compared with the CL in the female participants (*p* < .001, d = 0.85). The male IL showed greater increases compared with all other groups (*p* < .001–.037, d = 1.27–1.33). No differences were observed between the CLs of male and female participants (*p* = .987).

### 3.5 Measurement of hypertrophy

**FIGURE 8:**
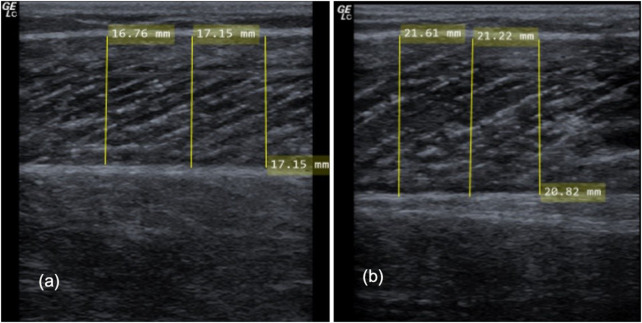
Illustrating the progression in muscle thickness (MTh) in the pre-test **(A)** and the post-test **(B)** exemplary.

For MThL there were significant increases associated with a time effect (F1,106 = 8.36, *p* = .005, ƞ^2^ = 0.073) but no significant Group*Time interaction (F3,106 = 0.95, *p* = .419, ƞ^2^ = 0.03). Figure 8 illustrates the pre-to post-test progression exemplary.

Statistical analysis showed time effect increases in MThM (F1,106 = 27.94, *p* < .001, ƞ^2^ = 0.21) and a Group*Time interaction (F3,106 = 7.31, *p* < .001, ƞ^2^ = 0.17). The Scheffé test revealed higher increases in the male IL compared with the female IL (*p* = .02, d = 0.51) but also compared with the CL groups (*p* < .001–.047 days = 0.46–0.72). No differences were observed between the female IL and CL (*p* = .63), as well as for the CL of the male and female participants (*p* = .416).

## 4 Discussion

The primary aim of this study was to investigate sex specific adaptations to long duration static stretch training in MSt, Mth and ROM in the plantar flexors showing significant higher increases for MVC180, KtW, ORTH and MThM in male participants compared with female participants. Results confirm significant baseline differences, stated in literature (Cipriani et al., 2012). Pre-test results showed significantly greater male MSt and higher female ROM, while no significant differences could be observed for Mth. However, relative values showed no significant difference for MVC180, while for MVC90 male participants showed higher MSt values. Concerning relative absolute training adaptations, males had greater responses in MVC180 (relative and absolute), KtW, ORTH and MThM compared to females.

### 4.1 Baseline differences between males and females

The absolute MSt values are in accordance with literature showing greater superiority for males compared to females (Nonaka et al., 2006; Nagai et al., 2020). The lack of significant MVC180 pre-test relative differences between sexes are in accordance with previous literature, suggesting that differences in strength capacity are trivial when normalized for fat-free mass (Freilich et al., 1995; Nonaka et al., 2006; Sandbakk et al., 2018). The present study showed no significant sex-related differences in the plantar flexors Mth. Furthermore, Abe et al. (Abe et al., 2021) demonstrated that females possess approximately 30% less muscle mass in the upper limbs compared to male participants; however, the magnitude of muscle mass seems to be more equal in the lower limbs. In contrast, Abe et al. (Abe et al., 2020) reported higher absolute muscle mass in trained and untrained male participants compared to females (Abe et al., 2003), which could potentially be attributed to sex-specific hormones influencing human collagen and muscles and therefore, subsequently, physical performance (Handelsman et al., 2018). Accordingly, Bishop et al. (Bishop et al., 1989) found no significant difference in arm muscle mass of trained swimmers, independent of sex, while in untrained participants males exhibited greater fat free mass. These findings suggest that the level of physical activity could be a more important factor influencing muscle mass and calls into question the high relevance of sex on muscle size. Furthermore, this raises the possibility that in non-athletic populations females have lower physical activity levels compared to males, thereby leading to significant differences in fat free muscle mass compared to male participants (Bishop et al., 1989). Since the present study included a very homogenous group of participants (moderately trained sport students and gym members), similar activity levels in this sample might be responsible for the lack of significant baseline calf MTh differences between male and female participants.

The flexibility results of the pre-test values were inconsistent in male and female participants. ORTH showed significantly higher pre-test ROM for females versus males, while no differences could be detected in KtW. Difference between tests might be the involvement of different knee angles. The KtW tested the ROM with a flexed knee, while using ORTH, the knee joint stayed extended, which influences the integration of different muscle fibers in the calf (Signorile et al., 2002; Arampatzis et al., 2006). Furthermore, Morrison et al. (Morrison et al., 2015) argued that Achilles tendon stiffness is primarily influenced by maximal strength. Assuming higher absolute male MSt values, the results can be seen as a confirmation of the hypothesis that higher stiffness and high MSt values seem to be linked, as MVC180 and MVC90 were significantly higher in males. Furthermore, Yu et al. (Yu et al., 2022) described a negative relationship between stiffness and flexibility. Therefore, if high MSt is linked to higher stiffness and lower flexibility, then lower flexibility accompanied by higher MSt in males could be expected (Cipriani et al., 2012; Yu et al., 2022). Furthermore, it can be speculated that there are more parameters influencing the ROM than flexibility of the muscle especially in the KtW test, e.g. joint- and bone structure, as an anteroposterior talocrural joint mobilization also led to significant changes in joint ROM, while no significant increases in flexibility of the muscle can be assumed (Holland et al., 2015).

### 4.2 Long-term effects of stretching

Results of this study, in addition to previous studies using animal and human models (Alway, 1994; Kelley, 1996; Warneke et al., 2022a; Warneke et al., 2022b; Warneke et al., 2022c), show that long duration static stretching routines could be considered as an alternative method to induce increases in MSt, MTh, and ROM in both sexes.

Most popular hypothesis explaining muscle hypertrophy effects and increases in MSt due to stretching attribute those responses to mechanical loading (Sola et al., 1973; Devol et al., 1991), resulting in structural damage of the fiber and triggering upregulation of anabolic signaling pathways such as mTOR, p70S6K (Aguilar-Agon et al., 2019; Boppart and Mahmassani, 2019) or calcineurin/calmodulin (Sakuma and Yamaguchi, 2010), which lead to enhanced protein synthesis (Sparrow, 1982; Czerwinski et al., 1994; Goldspink, 1999). In humans, it can also be assumed that high intensities in strength training lead to high mechanical loading and tension in muscular tissue, which can be hypothesized to be more effective to induce MSt compared with low load resistance training (Schoenfeld et al., 2015; Krzysztofik et al., 2019), while for MTh, the time of induced tension-stimulus and therefore the induced “time-tension-integral” seems to be also very important (Martineau and Gardiner, 2002; Schoenfeld et al., 2017). Accordingly, Kremer (Kremer, 2017) and Tegtbur et al. (Tegtbur et al., 2009) refer to stretching and strength training as sufficient stimuli to induce enhancements in protein synthesis *via* anabolic signaling pathways (Akt/mTOR/p70S6K) (Sasai et al., 2010; Suzuki and Takeda, 2011; Timur et al., 2019) in humans. To specify, it seems that mechanical tension per sarcomere could have a crucial impact on hypertrophy and MSt increases. Devol et al. (Devol et al., 1991) showed throughout the first days of training greater increases in muscle cross-sectional area. Afterwards, there was a decrease in hypertrophy, which can possibly be attributed to lower mechanical tension per sarcomere after few days of stretching without re-adjusting the stretching intensity by reaching higher degrees of stretch. In the study, the authors hypothesized the lower mechanical load per sarcomere due to an increased number of sarcomeres in series would lead to lower mechanical tension per contractile unit. Accordingly, the highest increases in muscle mass in animal studies (318%) were achieved when performing a progressively increasing stretching stimulus from 10% of the animal’s bodyweight to 35% over about 5 weeks (Antonio et al., 1993). If mechanical loading, irrespective of stretching or strength training, is of high impact on physiological responses in the muscle (Kjær, 2004; Tegtbur et al., 2009; Kremer, 2017), questions arise about sex differences, as factors such as IGF-1, HGH, FGF are present in both sexes. First, in males there are higher chronic values of testosterone, which seems to be important for adaptations of RT (Tipton, 2001). Secondly, it can be hypothesized that in females, it might be more difficult to induce high mechanical tension *via* stretching. Ryan et al. (Ryan et al., 2011) showed that passive stiffness and peak torque as well as passive stiffness and amount of muscle tissue are well correlated. Accordingly, lower strength capability and smaller muscle cross-section or muscle thickness might be related to lower stiffness, which might influence the passive peak torque and therefore, it can be hypothesized that it may be harder to reach high mechanical tension *via* stretch.

Especially considering the significant higher flexibility in females in the present study (i.e., in ORTH) and in previous research (Cipriani et al., 2012) with an accompanied lower degree of stiffness (Yu et al., 2022), it could be speculated that it is more challenging to induce a high mechanical stimulus to the stretched muscle compared to males. It might be hypothesized that in male participants, due to lower baseline flexibility, higher relative magnitudes of ROM improvements could be reached compared to females. Since high degrees of stretch are reported to be important to induce greater ROM and changes in muscle architecture (Apostolopoulos et al., 2015; Freitas et al., 2015; Freitas and Mil-Homens, 2015), it can be hypothesized that differences in stretch intensity could be responsible for sex differences.

However, since intensity in stretching is often quantified using the VAS (Freitas et al., 2015; Warneke et al., 2022b; Warneke et al., 2022c; Hatano et al., 2022) there is a lack of objective quantification of stretching intensity in the present and previous studies (Lim and Park, 2017), consequently, the role of stretching intensity remains hypothetical. Even if in some studies [such as Simpson et al. (Simpson et al., 2017)], a standardized weight was used to perform stretching, considering the passive torque as an important factor to achieve mechanical tension, quantification of intensity seems still questionable. Therefore, an objective quantification of intensity in stretching literature is necessary in further studies (Nakamura et al., 2021).

The hypothesis that sex-related differences would depend on the analysis of absolute vs. relative values (Jones et al., 2021) was not confirmed in this study. There were only small differences between stretching responses regarding MSt between absolute and relative values. It could be hypothesized that differences in the magnitude of adaptations between absolute and relative values might be a result of sex differences in bodyweight or fat free muscle mass. As the participants were all classified as athletically active, a comparably high homogeneity could be assumed within the group, so normalization of the data for body mass would only result in a homogeneous downshift of values without changing the relationships. Hence, changes in the measures due to stretching seem not to depend on taking body weight into account, which is in contrast with findings of Varley-Campbell et al. (Varley-Campbell et al., 2018) and Jones et al. (Jones et al., 2021). According to the literature (Sandbakk et al., 2018; Jones et al., 2021), males had higher absolute muscle mass in pre-as well as post-test compared to females in the calf muscle and higher MSt measured *via* MVC90.

Furthermore, the present study showed significant increases in MVC180 and MVC90 in the non-stretched contralateral leg, which served as a control condition in this study. Previous research showed significant contralateral force transfer as well (Nelson et al., 2012; Caldwell et al., 2019; Warneke et al., 2022b; Warneke et al., 2022c). Since the measurement design of the present study is limited through the absence of a non-intervened control group, interpretation regarding the contralateral force transfer is limited. However, previous research using similar groups of participants showed no significant increase in control conditions while significant increases in MSt in the contralateral control leg were reported as well. The contralateral force transfer is well known from strength training routines (Lee and Carroll, 2007; Manca et al., 2021). An explanatory approach of Zhou (2000, 2022) attributes increases in the contralateral leg with afferent signals induced by different training routines, which could be present without a central innervation of the muscle by voluntary contraction. This may be of an importance because no voluntary contraction can be assumed in passive stretch. The finding of significant increases in ROM for both sexes is common in the literature (Cipriani et al., 2012; Cejudo et al., 2020) and is generally attributed to changes in pain threshold or pain perception (Freitas and Mil-Homens, 2015; Freitas et al., 2018), or by changes in the muscle tendon unit architecture (Abellaneda et al., 2009; Nakamura et al., 2017).

### 4.3 Practical applications

Increases in MSt and MTh are commonly associated with resistance training routines. Results of this study are in accordance with previous findings of Warneke et al. (Warneke et al., 2022b; Warneke et al., 2022c). It can be hypothesized that the stretch training can possibly be used as a substitution for resistance training, especially if no active resistance training can be performed to increase MSt and MTh. This is, for example, the case in rehabilitation of immobilization-induced muscular atrophy and strength loss (Stevens et al., 2004; Wilson et al., 2019), if no joint stress or active muscle contraction can be induced by conventional resistance training. The individual could provide beneficial anabolic stress to the affected muscle while watching television, seated and working on a computer or other similar sedentary activities. Therefore, previous research pointed out an application of 1 h of stretch to counteract muscular imbalances in the calf muscles (Warneke et al., 2022d) without active muscle contraction. Therefore, the results (partially) confirm recent findings of Li et al. (Li et al., 2022) showing that in participants with low baseline strength values a sole flexibility training seems to be effective in increasing strength and flexibility capacity. However, results of the current study showing also that stretching with sufficient intensity and duration provide an appropriate stimulus to induce strength, flexibility and muscle thickness increases in trained participants. Therefore, the current findings provide deeper insights in this topic, showing that using stretching, effects are superior in male participants.

### 4.4 Limitations

This study had some limitations. Sonography is the most used assessment to investigate hypertrophy effects because of its relatively low cost and time-efficiency, and is a valid and reliable procedure to investigate MTh and MCSA (Mendis et al., 2010; Betz et al., 2021). Nevertheless, there are studies showing limitations of sonography especially because of subjective influence of pressure of the transducer and no real standardization (Hebert et al., 2009; English et al., 2012; Warneke et al., 2022e), which were confirmed in present study showing high %-SDs for changes in MTh of the control group. However, the ICC in the present study is very high for the obtained measures, providing confidence in the results. In addition, we did not endeavor to investigate the physiological mechanisms underlying the increases in range of motion. Thus, we cannot draw strong inferences as to the underlying explanatory causes of our findings. In general, adaptations might be influenced by sex hormones. Those were not assessed regarding this study, consequently, all discussion about this remains speculative.

## 5 Conclusion

There are previous studies investigating effects of long-duration stretch training on MSt, ROM and MTh in animals and humans, however, without consideration of sex-related differences. The present study showed that a daily stretching regimen of 1 hour over 6 weeks led to significant increases in MSt of up to 15% and 8%, ROM of up to 13% and 21% and MTh of up to 5% and 14% in male and female participants, respectively. However, increases were significantly higher in male participants compared with females in most measured parameters. Evaluation procedures in the current stretching literature typically assess stretching intensity by using subjective pain threshold, which seems to lack objective (quantitative) sensitivity. Consequently, further studies should include an objective quantification of intensity.

## Data Availability

The raw data supporting the conclusion of this article will be made available by the authors, without undue reservation.
